# Severe hypertension in pediatric diabetic ketoacidosis a case report and review of literature

**DOI:** 10.51866/cr.93

**Published:** 2022-11-22

**Authors:** Syed Ahmed Zaki, M Guftar Shaikh, Asrar Rashid

**Affiliations:** 1 MD, MRCPCH, Department of Paediatrics, All India Institute of Medical Sciences, Bibinagar Hyderabad, India. Email: drzakisyed@gmail.com; 2 MD, FRCPCH, Department of Endocrinology, Royal Hospital for Children, Glasgow, UK.; 3 MRCP, FRCPCH, Department of Paediatric intensive care unit, NMC Royal hospital, Abu Dhabi, UAE.

**Keywords:** Diabetic ketoacidosis, Hypertension, Dehydration, Hypovolaemia, Children

## Abstract

Diabetic ketoacidosis (DKA) is a life-threatening complication of type 1 diabetes mellitus in children. Despite the presence of dehydration, hypertension occurs in a significant proportion of children with DKA. There is a lack of clarity in the literature regarding the management of hypertension in patients with paediatric DKA. Herein, we report the case of an adolescent boy who presented with DKA and severe hypertension. His neurological status was closely monitored. There was a gradual decline in his blood pressure with an improvement in the pH over the next 72 hours. The combination of severe DKA and hypertension can be a challenging clinical dilemma, especially regarding fluid management. Studies on severe DKA in children are exacting, given the rarity of this condition. A multi-centre study is suggested to provide a meaningful analysis of this aspect of DKA.

## Introduction

Although preventable, diabetic ketoacidosis (DKA) remains the most severe complication in children with type 1 diabetes mellitus (DM).^1,2^ Fluid losses from osmotic diuresis, vomiting and hyperventilation in patients with DKA can lead to hypovolaemia and dehydration.^3^ Additionally, insulin deficiency increases the production of vasodilative prostaglandins.^4^ Thus, the expected haemodynamic response is tachycardia with a normal or low blood pressure (BP). In contrast, despite the presence of hypovolaemia and insulinopaenia, paradoxical hypertension in patients with severe paediatric DKA has been scatteredly reported.^2,5-7^ To date, data on severe hypertension secondary to paediatric DKA remain limited. We report the case of a child who presented with DKA and severe hypertension and present the findings of a review of the literature.

## Case presentation

A 14-year-old boy of Arab descent presented to our emergency department with a history of vomiting, abdominal pain, decreased oral intake for 3 days and breathlessness for 2 days. He was diagnosed with type 1 DM 3 years prior and was on basal-bolus subcutaneous insulin regimen (regular and long-acting injections). His HbA1c level assessed 3 months prior was 12.6%. On arrival at the emergency department, he was dehydrated (approximately 9%) with Kussmaul breathing. He was mildly agitated with a Glasgow Coma Scale (GCS) score of 15/15. His vital signs were as follows: heart rate, 152 beats/min; BP, 202/103 mmHg; respiratory rate, 32 cycles/min; and oxygen saturation level, 99% on room air. On examination, he had tenderness in the epigastric area. The other examination findings were normal. The bedside glucose level was high, with severe metabolic acidosis in venous blood gas analysis (**Table 1**). He was started on local DKA protocol and subsequently moved to the paediatric intensive care unit. His BP was recorded using an oscillometric device in the supine position with an appropriately sized arm cuff. The laboratory findings were suggestive of severe DKA (**Table 1**).

The renal Doppler ultrasound and 12-lead echocardiogram findings and cardiac enzyme levels were normal. Generally, children with DKA have poor peripheral perfusion, and cerebral perfusion depends on a high peripheral vascular resistance. Thus, antihypertensive medications may precipitate hypotension, thereby worsening the neurological status. Owing to a lack of clarity in the literature regarding the pathophysiological process of DKA-associated hypertension and the risk of cerebral oedema (CE), a policy of close neurological monitoring without active treatment of hypertension was instituted. The neurological status was strictly monitored in accordance with the ISPAD Clinical Practice Consensus Guidelines 2018 for paediatric DKA management.^1^ The hydration status was assessed on the basis of the heart rate, peripheral perfusion, BUN level and haematocrit level. Intake and output were also strictly monitored. With the initiation of fluid resuscitation in the emergency department, there was a gradual reduction in his BP. The simultaneous improvement in the BP and pH continued over the next 72 hours. The boy was mildly agitated 4 hours after admission and remained somnolent for the next 24 hours. His GCS score remained 15/15 throughout the hospital course. His BP returned to normal after 72 hours, and he was discharged after 4 days of hospital stay. On follow-up visits, there were no clinical concerns regarding his neurological state and BP

**Table 1:** Laboratory findings during hospital stay.

Day	1	1	1	1	2	2	3
Time after admission (h)	0	2	12	24	36	48	72
Urea level (mmol/L)	9.17	-	-	6.2	3.5	-	1.79
Serum creatinine level (mmol/L)	106.1	-	-	64	56	-	50
Serum sodium level (mmol/L)	127	-	136	141	142	140	142
Serum potassium level (mmol/L)	6.7	-	3.46	3.6	3.7	3.8	3.6
Chloride level (mmol/L)	98.8		110	107	114	112	110
Venous pH	6.755	6.9	7.005	7.209	7.215	7.27	7.32
pCO_2_ level (mmHg)	19	24	24	28.8	31	34	36
HCO_3_ level (mmol/L)	2.7	4	5.4	11.2	13.5	15.2	19
Base excess level	-32.6	-29	-27.8	-15.2	-14.2	-10.4	-8.4
Urinary ketone	4+	4+	-	1 +	-	nil	-
Blood pressure (mmHg)	202/103	184/94	155/89	147/83	139/74	127/68	132/74
Glucose level (mmol/L)	32		21	12.5	10.8	9.7	8
Renal ultrasound findings	Normal						
Cardiac enzyme levels	Normal						

## Discussion

Deeter et al. found that despite the presence of severe dehydration, no children with DKA had hypotension. On the contrary, 48% were found to have hypertension on admission, and 82% developed such within 6 hours of admission.^6^ Similarly, DePiero et al. reported hypertension in 12.2% of patients, with an additional 15.6% of patients developing such during treatment. They found that the presence of severe acidosis, a lower pCO_2_ level and a lower GCS score were significantly associated with hypertension.^7^ Other risk factors were present in our patient, except for the low GCS score. Although the international guidelines for paediatric DKA mentions hypertension, guidance for its management is lacking.^1^ Further, the available literature on DKA-associated hypertension in children is limited.^6,7^ Thus, clinicians must rely on their clinical experience in managing hypertension in patients with paediatric DKA. Despite the presence of dehydration, hypertension likely reflects complex systemic pathophysiological processes that may be unique to DKA. Various theories have been put forth to explain paradoxical hypertension in individuals with DKA. These include overactivity of the renin—angiotensin—aldosterone system (RAAS), sympathetic nervous system and vasopressin system.^2,5-7^ Low plasma levels of atrial natriuretic peptide (ANP) are seen in patients with severe DKA due to hypovolaemia. ANP inhibits the vasoconstrictor action of norepinephrine and decreases the secretion of vasopressin, renin and aldosterone. Thus, low levels of ANP can result in an unopposed action of these hormones, leading to hypertension.^2,5-7^ Antidiuretic hormone (ADH) release due to hyperosmolality and volume depletion may also increase the BP via V2 receptors and increase the peripheral vascular resistance.^6^ Hypovolaemia is responsible for the initial activation of the RAAS, ANP and ADH. In addition, the associated severe acidosis stimulates stress reactions, thereby activating the compensatory mechanisms. This increases the production of counter-regulatory hormones (e.g. glucagon, cortisol and growth hormone) and proinflammatory cytokines in patients with DKA, which can lead to hypertension.^2,5-7^ Restoring the fluid status and correcting acidosis will ameliorate some of these mechanisms and help in reducing the BP.

Studies have also documented abnormalities in cerebral blood flow during DKA, resulting in altered brainstem perfusion, which may interfere with the autoregulatory control mechanisms of the BE^8^ We hypothesised that one or more of the above-indicated mechanisms may have resulted in hypertension in our patient. Further prospective studies are needed to confirm or refute this hypothesis. Owing to financial constraints, we could not evaluate the plasma levels of all above-mentioned hormones. Another possibility underlying the development of hypertension in patients with paediatric DKA is CE. It occurs in 0.2–1% of patients and is a potentially severe complication with high morbidity and mortality.^1,9^ However, early detection of CE is notoriously difficult. Severe CE can increase the intracranial pressure, leading to systemic hypertension. Additionally, the stress response to CE may lead to increased catecholamine levels, resulting in hypertension.^5^ The pathophysiological understanding of CE in patients with DKA remains controversial. Various theories include rapid fluid administration, abrupt changes in serum osmolality, dehydration and cerebral hypoperfusion.^1,9^ Thus, deciding to intervene is challenging yet crucial, as early treatment can prevent adverse neurological outcomes. There can be reluctance to volume expansion in children with DKA and hypertension for fear of worsening hypertension. Further, it is not prudent to use antihypertensives, as these patients have poor peripheral perfusion.^1^ Additionally, cerebral perfusion may be dependent on a high peripheral vascular resistance. Thus, antihypertensive medications may precipitate hypotension, thereby worsening the neurological status. Until the pathophysiology of DKA-associated hypertension is fully understood, a conservative approach and adherence to the ISPAD fluid guidelines are suggested. A gradual decrease in the BP with an improvement in pH and dehydration, as in our case, would indicate that the treatment is appropriate. We believe such a strategy minimises the risk of complications and could ameliorate hypertension, as seen in our patient. However, during this period of fluid resuscitation, close monitoring of the neurological status is of utmost importance.^1^ Muir et al. suggested an evidence-based protocol for early detection of CE at the bedside, which permits timely intervention to prevent permanent brain damage. Neuroimaging is not required for the diagnosis of CE.^1,9^

## Conclusion

Hypertensive crises can occur in patients with severe paediatric DKA and complicate the fluid resuscitation strategy. The BP should not be used as a marker to assess the degree of dehydration in individuals with DKA. Close monitoring of the neurological status during treatment is essential. A multi-centre study is suggested to provide a meaningful analysis of the pathophysiology of hypertension in patients with severe DKA. Finally, international guidelines should provide direction on the management of hypertension in these patients.
